# Application of the ALBI Scoring System for Mortality Outcome Prediction in Patients with Hypertrophic Cardiomyopathy

**DOI:** 10.5334/gh.1163

**Published:** 2022-10-11

**Authors:** Ping Qiao, Liying Li, Haiyan Ruan, Muxin Zhang, Ziqiong Wang, Xinran Li, Rufeng Shi, Xin Wei, Linjia Duan, Yi Zheng, Sen He

**Affiliations:** 1 Department of Cardiology, West China Hospital of Sichuan University, Chengdu, China; 2 West China School of Nursing/West China Hospital, Sichuan University, Chengdu, China; 3 Department of Cardiology, Hospital of Traditional Chinese Medicine, Shuangliu District, Chengdu, China; 4 Department of Cardiology, First People’s Hospital, Longquanyi District, Chengdu, China; 5 Department of Cardiology and National Clinical Research Center for Geriatrics, West China Hospital of Sichuan University, Chengdu, China

**Keywords:** albumin, bilirubin, mortality, hypertrophic cardiomyopathy

## Abstract

**Background:**

There is no information about the clinical significance of the albumin-bilirubin (ALBI) score in patients with hypertrophic cardiomyopathy (HCM).

**Objective:**

We retrospectively performed clinical evaluations in 462 patients with HCM to estimate whether the ALBI score could be a new tool to predict mortality in HCM.

**Methods and Results:**

During a median follow-up of 4.7 years, HCM-related death occurred in 52 (11.3%) patients. Overall, there was a significant positive association between ALBI score and HCM-related death (adjusted hazard ratio [HR]: 1.79 per one standard deviation [SD] increment, 95% confidence interval [CI]: 1.36–2.35). When the score was assessed as tertiles, the adjusted HRs of HCM-related death were 1.30 (95% CI: 0.42–3.99) for the tertile 2 and 4.43 (95% CI: 1.65–11.89) for the tertile 3, compared with the tertile 1. Stratified analysis and E-value analysis suggested the robustness of the above-mentioned results. Meanwhile, time-dependent ROC analysis showed ALBI score could discriminate HCM-related death at various time points (AUC ranges: 0.725–0.850). Furthermore, exploratory analysis indicated the dynamic changes of ALBI score also could predict HCM-related death. Finally, multiple linear regression analysis suggested some pathogenetic pathways associated with HCM-related adverse outcomes significantly correlated with ALBI score, and the pathways included inflammation, myocardial injury, nutritional status and some clinical characteristics, but not abnormal cardiac structure and function itself.

**Conclusions:**

Higher ALBI score is a strong independent predictor of HCM-related death in patients with HCM.

## 1. Introduction

Hypertrophic cardiomyopathy (HCM) is a genetic heart disease that affects patients globally, with an estimated prevalence of at least 1:500 [1]. Innovative clinical science over the past two decades has greatly improved life expectancy and the quality of life for patients with HCM [2]. However, recent studies suggest that HCM is still associated with excess mortality [3,4,5]. Therefore, we need more researches about the risk associated with HCM-related death and for better risk stratification in patients with HCM.

Recently, a new scoring system assessing liver function, namely albumin-bilirubin (ALBI) score, was developed in patients with hepatocellular carcinoma [6], and many studies have shown that higher ALBI score is associated with adverse events in various gastrointestinal diseases [7,8,9,10]. Meanwhile, it is well established that liver function can predict the prognosis in patients with cardiovascular diseases (CVDs) [11,12,13]. However, only two studies assessed the relationship between ALBI score and CVDs: one showed the score was independently associated with in-hospital mortality in patients hospitalized for acute heart failure (HF) [14], and another suggested it could predict one-year mortality in patients with acute HF [15]. On the other hand, recent researches have demonstrated the two components of ALBI score, total bilirubin and albumin, are associated with inflammation and oxidative stress [13,16,17], which are related to ventricular remodeling, myocardial fibrosis, diastolic dysfunction, microvascular thrombosis, cardiac hypertrophy and left ventricular outflow tract obstruction in HCM [18,19,20,21,22]; meanwhile, total bilirubin and albumin also have some other properties [12,17], which could be associated with the prognosis in patients with HCM. Therefore, ALBI score may a potential prognostic indicator for HCM.

To our knowledge, there is no information about the clinical significance of ALBI score in patients with HCM. Therefore, we tested the hypothesis that higher ALBI score was associated with increased risk of death in patients with HCM.

## 2. Methods

### 2.1. Study patients

This was a retrospective, single-center cohort study, which was performed at West China Hospital of Sichuan University, and the hospital is a tertiary center located in Chengdu, China. We included the hospitalized patients with a discharge diagnosis of HCM (n = 546) between December 2008 to November 2018, and then, the diagnosis was rechecked by experienced cardiologist according to the criteria of the European Society of Cardiology (ESC) [23]. After rechecking, nine patients were excluded for cardiac amyloidosis (n = 5), restrictive cardiomyopathy (n = 2), dilated cardiomyopathy (n = 1) and myocarditis (n = 1). Among the remaining 537 patients, 72 patients with missing baseline data (n = 67) or age under 18 (n = 5) were excluded from the study, and 3 patients were also excluded from the study because of loss to follow-up after the first evaluation. Finally, a total of 462 adult patients were included for the present analysis.

The study was conducted according to the principles of the Declaration of Helsinki, and was approved by the Biomedical Research Ethics Committee, West China Hospital of Sichuan University (approval number: 2019–1147). The study has been registered on the website of Chinese Clinical Trial Registry (https://www.chictr.org.cn/enIndex.aspx; registration number: ChiCTR2000029352). Informed consent was waived due to the retrospective nature of the study. Some other detailed information has been reported in the recently published studies [24,25,26].

### 2.2. Clinical evaluation

Baseline characteristics were collected from medical records by experienced physicians, and these characteristics mainly included medical histories, therapies, Doppler echocardiography, peripheral blood parameters and 12-lead electrocardiogram. The twice-entry method was used for data entry. When the values of the two entries were consistent, the data would enter the database; otherwise, the raw data would be checked.

In our clinical medical laboratory center, which is accredited by the American CAP Medical Laboratory, normal ranges of total bilirubin (TBil) and serum albumin are 5.0–28.0 μmol/L and 35–55 g/L, respectively. Based on the literature [6], ALBI score was calculated using the following formula: ALBI score = 0.66*log_10_TBil (μmol/L) – 0.085*albumin (g/L). Patients were divided into three groups according to the tertiles of baseline ALBI score: tertile 1 (< –3.01), tertile 2 (–3.01 to < 2.71) and tertile 3 (>= –2.71).

### 2.3. Study outcome

On the basis of the previous studies [27,28,29], the common modes of HCM-related death include: HF-related death, stroke-related death and sudden cardiac death (SCD). Therefore, the study outcome was defined as a composite of the above-mentioned three modes of HCM-related death and other specific HCM-related death. Specially, HF-related death was defined as death preceded by signs and/or symptoms of HF of more than one hour duration, which was in the context of progressive cardiac decompensation more than one year before death [30]. Stroke-related death was defined as a result of probable or proven ischemic stroke [31], without distinguishing cardioembolic stroke from other ischemic subtypes in the present study. SCD was defined as witnessed sudden cardiac death within one hour of new symptoms or nocturnal death in patients who previously experienced a relatively stable or uneventful clinical course [32]. Other specific HCM-related death was defined as death due to HCM, but not in the aforementioned three conditions.

Patients were followed up via telephone interviews or medical records, and followed from the initial evaluation up to the outcomes or the most recent evaluation.

### 2.4. Statistical analysis

We conducted analyses with the following steps: (1) comparison of descriptive data at baseline; (2) determination of risk-adjusted estimates for HCM-related death, as well as the robustness of risk-adjusted estimates by stratified analysis in various subgroups and E-value analysis, and evaluating accuracy of ALBI score in discriminating HCM-related death at different time points; (3) comparisons of ALBI score versus other parameters of liver function and the HCM risk-SCD score in predicting HCM-related death; (4) exploring whether serial determinations of ALBI score could also predicting HCM-related death in 208 patients who had the second measurement of albumin and TBil. Changes in ALBI score from baseline to the second measurement were expressed by categorical changes and based on the changes in ALBI score across a threshold level (median ALBI score: –2.8), patients were divided into four groups: low-low (patients with ALBI score below the threshold at baseline and the second measurement); low-high (patients with ALBI score below the threshold at baseline and above at the second measurement); high-low (patients with ALBI score above the threshold at baseline and below at the second measurement); high-high (patients with ALBI score above the threshold at baseline and the second measurement); (5) exploring analysis the potential mechanisms why ALBI score could predict HCM-related death.

By the tertiles of ALBI score, continuous variables were presented as mean ± standard deviation or median with interquartile range (IQR) where appropriate, and number (percentage) for categorical variables. For continuous variables, p-value for trend across the three groups was computed from the Pearson test when row-variable was normal distribution and from the Spearman test when it was non-normal distribution. When the row-variable was categorical, p-value for trend was computed from Mantel-Haenszel test of trend.

Survival curves were constructed using Kaplan-Meier estimates, and HCM-related death rates were compared using the log-rank test. For prognostic analysis, Cox proportional hazard regression model was applied. Baseline variables that showed a univariate relationship with HCM-related death (p < 0.05) were entered into the following multivariate Cox regression models 1–4 to evaluate the power of ALBI score predicting HCM-related death from different perspectives: (1) model 1, the basic model, included age and gender; (2) model 2 included the basic model and clinical parameters; (3) model 3 extended model 2 plus laboratory values; (4) model 4 included model 3 and echocardiographic data. For the final model 5, variables for inclusion were carefully chosen, given the number of events available, to ensure parsimony of the final model. The predictors of model 5 were sought using a backward stepwise modeling approach including all variables from models 1 to 4 (except ALBI score). Based on the literature [33], available variables associated HCM-related death in our study were also included in the final model, even if there was no statistical significance in this study. Furthermore, the robustness of the above-mentioned results was assessed by stratified analysis and E-value analysis. Specially, E-value analysis could assess the potential for unmeasured confounding between ALBI score and HCM-related death, and it quantifies the required magnitude of an unmeasured confounder that could negate the observed association between ALBI score and HCM-related death [34]. Meanwhile, a time-dependent receiver operating characteristic (ROC) curve was generated to evaluate the accuracy of ALBI score in discriminating HCM-related death at different time points. A generally accepted approach suggests that an area under the ROC curve (AUC) of less than 0.60 reflects poor discrimination; 0.60 to 0.75, possibly helpful discrimination; and more than 0.75, clearly useful discrimination [35].

Compared with ALBI score, we assessed some other parameters of liver function in predicting HCM-related death, and also evaluated the relationships between HCM risk-SCD score and the study outcome. In addition, we performed exploratory analysis to evaluate whether the changes of ALBI score during the follow-up could predict HCM-related death. Finally, the relationship between ALBI score and other indicators, which have been associated with HCM-related adverse outcomes, was analyzed to explore the potential mechanisms why the score could predict HCM-related death.

All analyses were performed with R version 4.1.0 (R Project for Statistical Computing) including the ‘compareGroups’, ‘rms’, ‘survminer’, ‘tidyverse’, ‘survival’, ‘timeROC’, ‘survivalROC’, ‘forestplot’ and ‘stats’ packages. All tests were two sided, and p values < 0.05 were considered statistically significant.

## 3. Results

### 3.1. Baseline characteristics

The present study comprised 462 patients (male: 54.76%) with a median age of 58.00 (IQR: 46.00–67.00) years. ALBI score ranged from –3.83 to –1.05 (median: –2.88, IQR: –3.11 to –2.61). Baseline characteristics of the three groups by the tertiles of ALBI score are summarized in Table 1. ALBI score was positively associated with age, NYHA III-IV, prior thrombo-embolic event, atrial fibrillation, TBil and neutrophil to lymphocyte ratio (NLR) at baseline; and were inversely associated with taking beta blockers, albumin, triglyceride (TG), low density lipoprotein cholesterin, hemoglobin and lymphocyte count at baseline, as well as left ventricular ejection fraction.

**Table 1:** Population characteristics by tertiles of baseline ALBI score.


VARIABLE	ALL	ALBI SCORE			p VALUE FOR TREND

		TERTILE 1 (< –3.01)	TERTILE 2 (–3.01 to < 2.71)	TERTILE 3 (>= –2.71)	

No. of patients (n)	462	151	152	159	

Gender, male	253 (54.76%)	89 (58.94%)	76 (50.00%)	88 (55.35%)	0.540

Age (years)	58.00 (46.00, 67.00)	53.00 (42.00, 66.00)	56.00 (46.75, 66.00)	62.00 (49.00, 70.50)	<0.001

Family history of HCM	40 (8.66%)	16 (10.60%)	12 (7.89%)	12 (7.55%)	0.344

Family history of SCD	15 (3.25%)	6 (3.97%)	6 (3.95%)	3 (1.89%)	0.297

NYHA III-IV	161 (34.85%)	47 (31.13%)	40 (26.32%)	74 (46.54%)	0.004

Symptom					

Dyspnea	257 (55.63%)	72 (47.68%)	92 (60.53%)	93 (58.49%)	0.059

Chest pain	248 (53.68%)	84 (55.63%)	87 (57.24%)	77 (48.43%)	0.199

Syncope/pre-syncope	144 (31.17%)	44 (29.14%)	56 (36.84%)	44 (27.67%)	0.760

Palpitation	174 (37.66%)	55 (36.42%)	61 (40.13%)	58 (36.48%)	0.999

Medical history					

Prior thrombo-embolic event	22 (4.76%)	4 (2.65%)	3 (1.97%)	15 (9.43%)	0.005

Vascular diseases	37 (8.01%)	9 (5.96%)	13 (8.55%)	15 (9.43%)	0.263

Hypertension	147 (31.82%)	54 (35.76%)	52 (34.21%)	41 (25.79%)	0.058

diabetes	39 (8.44%)	10 (6.62%)	12 (7.89%)	17 (10.69%)	0.197

Atrial fibrillation	82 (17.75%)	16 (10.60%)	25 (16.45%)	41 (25.79%)	<0.001

Hepatic disease	41 (8.87%)	10 (6.62%)	14 (9.21%)	17 (10.69%)	0.209

Therapy					

Aspirin	85 (18.40%)	34 (22.52%)	24 (15.79%)	27 (16.98%)	0.215

Clopidogrel	28 (6.06%)	9 (5.96%)	10 (6.58%)	9 (5.66%)	0.908

Beta blockers	334 (72.29%)	119 (78.81%)	113 (74.34%)	102 (64.15%)	0.004

ACEI or ARB	92 (19.91%)	30 (19.87%)	32 (21.05%)	30 (18.87%)	0.821

Intervention of obstruction					

none	410 (88.74%)	132 (87.42%)	133 (87.50%)	145 (91.19%)	0.649

alcohol septal ablation	45 (9.74%)	18 (11.92%)	17 (11.18%)	10 (6.29%)	

septal myectomy	7 (1.52%)	1 (0.66%)	2 (1.32%)	4 (2.52%)	

Device					

none	406 (87.88%)	135 (89.40%)	134 (88.16%)	137 (86.16%)	0.390

pacemaker	21 (4.55%)	7 (4.64%)	5 (3.29%)	9 (5.66%)	

ICD	35 (7.58%)	9 (5.96%)	13 (8.55%)	13 (8.18%)	

Hematological result					

ALT (IU/L)	22.00 (16.00, 34.00)	23.00 (17.00, 37.50)	21.00 (14.00, 29.25)	23.00 (16.00, 36.50)	0.482

AST (IU/L)	26.00 (21.00, 33.00)	26.00 (22.00, 32.00)	24.00 (19.00, 29.25)	27.00 (21.50, 38.00)	0.135

TBil (μmol/L)	12.35 (9.03, 17.70)	10.90 (8.50, 13.35)	12.15 (9.07, 16.92)	15.00 (10.40, 21.10)	<0.001

Albumin (g/L)	42.30 (39.50, 45.00)	46.10 (44.60, 47.70)	42.40 (41.60, 43.50)	38.40 (35.50, 40.00)	<0.001

Creatinine (μmol/L)	80.10 (67.00, 94.55)	81.00 (68.50, 92.50)	76.00 (65.00, 92.00)	81.50 (68.10, 100.75)	0.314

Urea nitrogen (mmol/L)	6.04 (5.02, 7.78)	6.01 (5.18, 7.13)	5.80 (4.82, 7.38)	6.41 (5.10, 8.72)	0.072

TG (mmol/L)	1.25 (0.94, 1.87)	1.57 (1.08, 2.28)	1.29 (0.99, 1.79)	1.04 (0.80, 1.40)	<0.001

HDL-C (mmol/L)	1.27 (1.03, 1.55)	1.26 (1.02, 1.58)	1.30 (1.08, 1.54)	1.24 (0.96, 1.54)	0.474

LDL-C (mmol/L)	2.41 (1.83, 2.90)	2.51 (1.91, 2.99)	2.54 (1.92, 2.95)	2.23 (1.77, 2.83)	0.026

Hgb (g/L)	136.04 (21.39)	141.14 (19.70)	134.88 (19.39)	132.31 (23.81)	<0.001

WBCC (10^9^/L)	6.32 (5.20, 7.78)	6.60 (5.36, 7.69)	6.19 (5.28, 7.56)	6.12 (4.99, 8.21)	0.152

Neutrophils count (10^9^/L)	3.91 (3.04, 5.23)	4.09 (3.21, 5.19)	3.77 (3.04, 5.03)	3.90 (2.95, 6.01)	0.786

Lymphocyte count (10^9^/L)	1.61 (1.26, 1.98)	1.80 (1.44, 2.27)	1.61 (1.31, 1.95)	1.42 (1.04, 1.84)	<0.001

NLR	2.30 (1.70, 3.60)	2.20 (1.60, 3.25)	2.30 (1.70, 3.42)	2.80 (1.90, 4.15)	<0.001

Echocardiographic					

LVEDD (mm)	43.00 (40.00, 46.75)	44.00 (40.00, 47.00)	42.50 (40.00, 46.00)	42.00 (38.50, 47.00)	0.054

LA diameter (mm)	40.00 (35.00, 45.00)	40.00 (35.00, 44.00)	39.00 (35.00, 45.00)	40.00 (36.00, 45.50)	0.345

MWT (mm)	19.00 (17.00, 22.00)	19.00 (17.00, 22.00)	19.00 (16.00, 22.00)	19.00 (17.00, 21.00)	0.501

LVEF (%)	68.00 (63.00, 72.00)	69.00 (64.00, 73.00)	68.50 (64.75, 72.00)	68.00 (62.00, 71.00)	0.003

Resting LVOTG >= 30 mm Hg	198 (42.86%)	62 (41.06%)	74 (48.68%)	62 (38.99%)	0.694


*Note*: Values are mean ± SD, median (IQR) or n (%). *Abbreviations*: ACEI = angiotensin-converting enzyme inhibitor, ALBI = albumin-bilirubin, ALT = alanine aminotransferase, ARB = angiotensin receptor blocker, AST = aspartate aminotransferase, HCM = hypertrophic cardiomyopathy, HDL-C = high density lipoprotein cholesterin, Hgb = hemoglobin, ICD = implantable cardioverter defibrillator, LA = left atrial, LDL-C = low density lipoprotein cholesterin, LVEDD = left ventricular end-diastolic dimension, LVEF = left ventricular ejection fraction, LVOTG = left ventricular outflow tract gradient, MWT = maximal left ventricular wall thickness, NLR = neutrophil to lymphocyte ratio, NYHA = New York Heart Association, SCD = sudden cardiac death, SD = standard deviation, TBil = total bilirubin, TG = triglyceride, WBCC = white blood cell count.

### 3.2. Association between ALBI score and HCM-related death

Patients were followed for a median period of 4.7 years (IQR: 2.1–6.8 years; total person-years [PYs]: 2157.9). During the follow-up, 52 patients (11.3%) reached the outcome of HCM-related death, including 26 HF-related deaths, 10 stroke-related deaths, 14 SCDs and 2 HCM-related postoperative deaths. The overall mortality rate was 2.4 (95% CI: 1.8–3.1) per 100 PYs.

**Figure 1:**
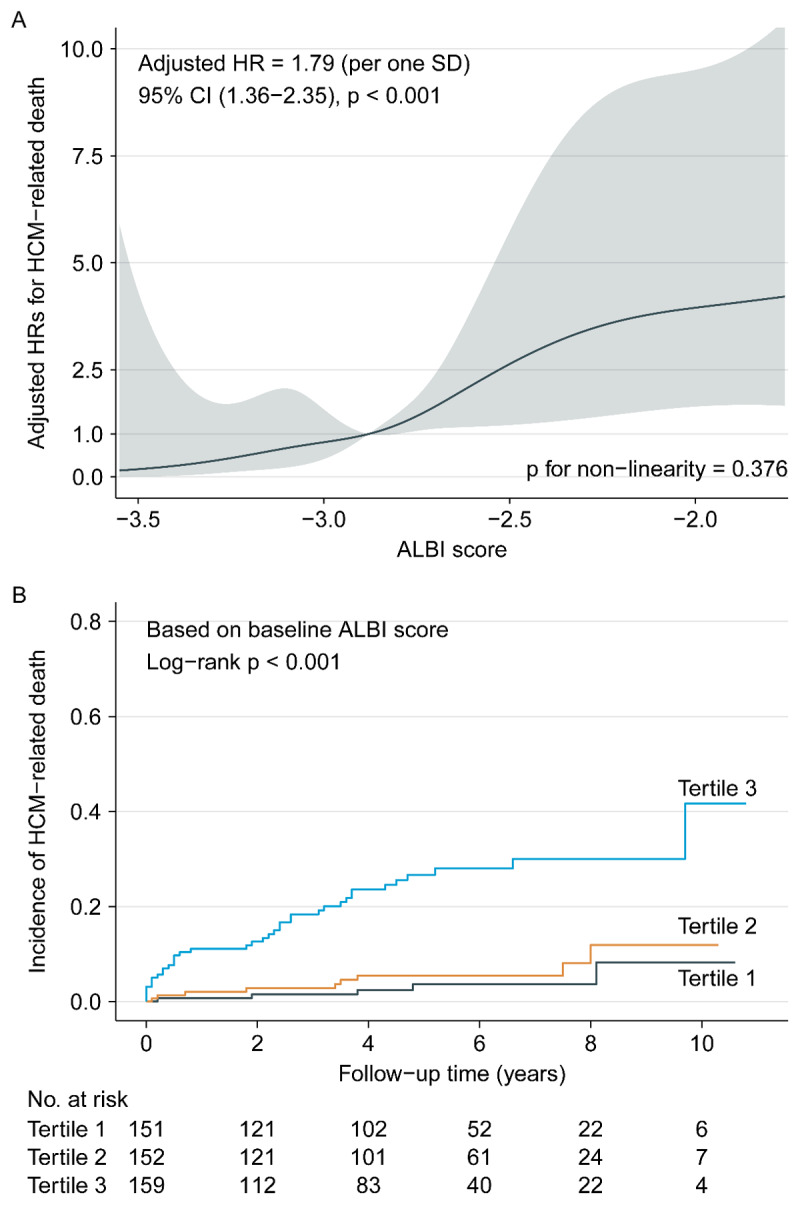
Association between ALBI score and HCM-related death. *Note*: **(A)** smooth curve fitting. HR was adjusted for age, gender, family history of SCD, NYHA III-IV, dyspnea, syncope/pre-syncope, atrial fibrillation, AST, urea nitrogen, TG, NLR, LA diameter, MWT and Resting LVOTG >= 30 mm Hg. The solid line and ribbon depict the HR and 95% CI, respectively. **(B)** Kaplan-Meier curves. Abbreviations as in Tables 1 and 2.

Overall, there was a significant positive association between ALBI score and HCM-related death (adjusted HR: 1.79 per one SD increment, 95% CI: 1.36–2.35, p < 0.001) (Figure 1A). When ALBI score was assessed as tertiles, Kaplan-Meier curve showed the clinical course of HCM-related death was significantly worse in the higher tertile (log-rank p < 0.001, Figure 1B). To assess whether the abovementioned relationship might be caused by chance, we further evaluated the relationship between ALBI score and some specific modes of HCM-related death, and the similar results were also found (Figures S1 and S2). Univariate Cox regression analysis indicated that ALBI score and some other variables could predict HCM-related death (Table 2 and Table S1). With the tertile 1 as reference, adjusted HRs for HCM-related death were 1.30 for the tertile 2 (95% CI: 0.42–3.99, p = 0.648) and 4.43 for the tertile 3 (95% CI: 1.65–11.89, p = 0.003), respectively (Table 2).

**Table 2:** Associations of ALBI score with HCM-related death.


	ALBI SCORE		

	TERTILE 1 (< –3.01)	TERTILE 2 (–3.01 to < 2.71)	TERTILE 3 (>= –2.71)

No. of patients (n)	151	152	159

HCM-related death (n)	5	9	38

HF-related death	2	2	22

Stroke-related death	1	2	7

SCD	1	5	8

HCM-related postoperative death	1	0	1

Follow-up time (PYs)	745	763.0	649.9

Mortality rate for HCM-related death (95% CI)⁎	0.7 (0.1–1.3)	1.2 (0.4–1.9)	5.8 (4–7.7)

Unadjusted HR for HCM-related death (95% CI), p	1	1.78 (0.60–5.32), 0.300	8.35 (3.28–21.22), <0.001

Adjusted HR for HCM-related death (95% CI), p			

Model 1	1	1.73 (0.58–5.17), 0.329	8.14 (3.16–20.94), <0.001

Model 2	1	1.66 (0.55–5.01), 0.368	5.96 (2.28–15.55), <0.001

Model 3	1	1.27 (0.41–3.90), 0.674	4.45 (1.66–11.99), 0.003

Model 4	1	1.27 (0.42–3.89), 0.673	4.28 (1.59–11.50), 0.004

Model 5	1	1.30 (0.42–3.99), 0.648	4.43 (1.65–11.89), 0.003


*Note*: Model 1 with adjustment for age and gender. Model 2 with adjustment for model 1 plus clinical parameters, including NYHA III-IV, dyspnea, prior TE and atrial fibrillation. Model 3 with adjustment for model 3 plus laboratory results, including AST, urea nitrogen, TG and NLR. Model 4 with adjustment for model 4 plus echocardiographic parameters, including LA diameter and LVEF. Model 5 with adjustment for age, gender, family history of SCD, NYHA III-IV, dyspnea, syncope/pre-syncope, atrial fibrillation, AST, urea nitrogen, TG, NLR, LA diameter, MWT and Resting LVOTG >= 30 mm Hg. ⁎Per 100 PYs. *Abbreviations*: CI = confidence interval, HRs = hazard ratios, PYs = person-years, other abbreviations as in Table 1.

**Figure 2:**
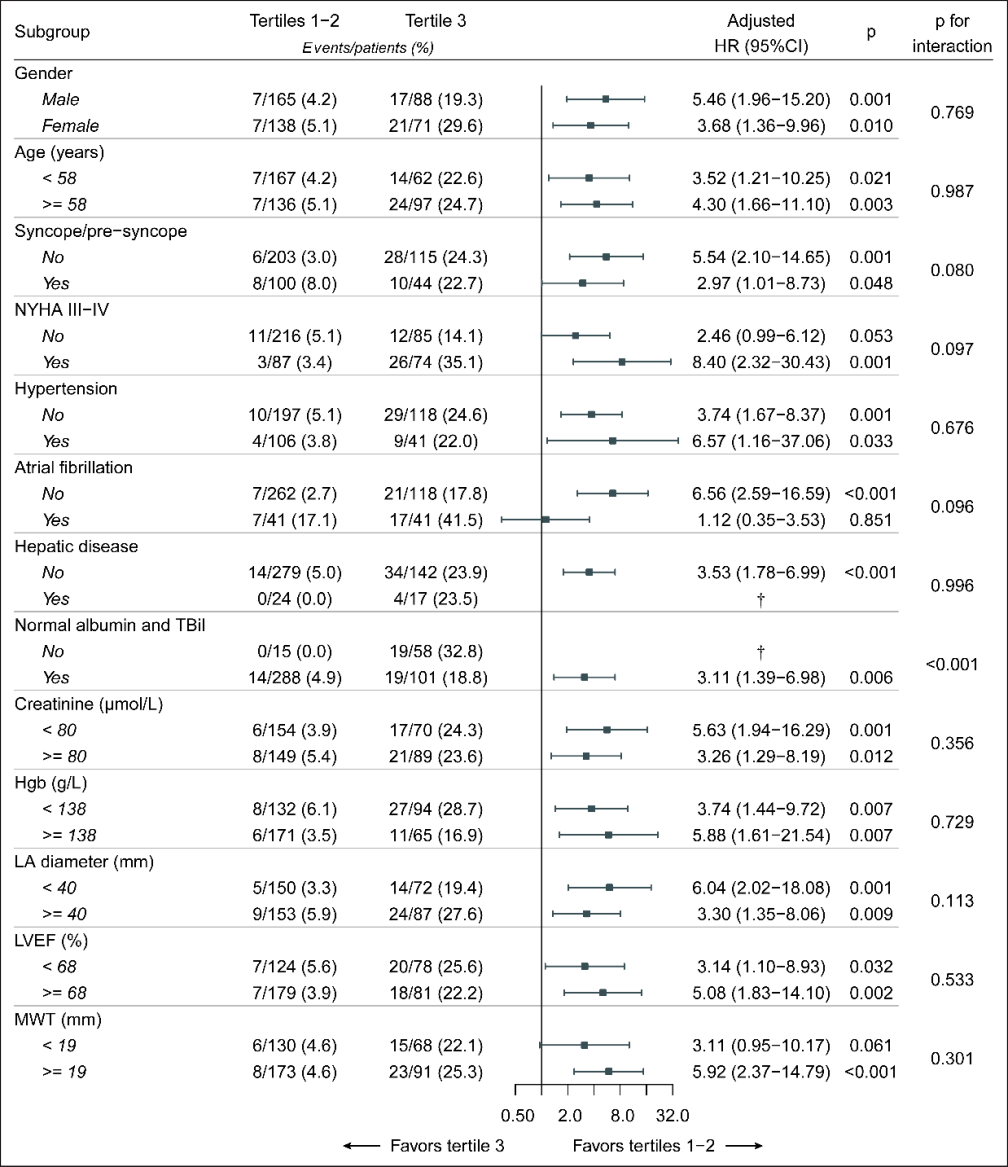
Stratified analyses of HCM-related death. *Note*: each stratification was adjusted for age, gender, family history of SCD, NYHA III-IV, dyspnea, syncope/pre-syncope, atrial fibrillation, AST, urea nitrogen, TG, NLR, LA diameter, MWT and Resting LVOTG >= 30 mm Hg, except the stratification factor itself. Grouping criteria of continuous variables were based on the median values. The p value for interaction represents the likelihood of interaction between variable and ALBI score. † The analyses failed because of no death in the tertiles 1–2. In addition, hepatic diseases included viral hepatitis (n = 10), alcoholic hepatitis (1), fatty liver (n = 8), diseases of the biliary system (n = 16), liver cirrhosis (n = 2) and unknown reasons (n = 4). Abbreviations as in Tables 1 and 2.

Due to a similar incidence in HCM-related death, we combined the patients in the tertiles 1 and 2 to construct a new group, namely tertiles 1–2. Then, we further performed stratified analysis in various subgroups (tertile 3 vs. tertiles 1–2) to assess the relationship between ALBI score and the risk of HCM-related death. The results showed that the mortality risk was consistently higher in the tertile 3 than in the tertiles 1–2 in all subgroups (Figure 2). In addition, the E-values for the effects of ALBI score on HCM-related death were 8.33 (lower limit of CI: 2.69) for the tertile 3 and 2.98 (lower limit of CI: 2.06) for the increment of one SD, respectively. This suggested that the main findings should be robust, unless an unmeasured confounder existed with a higher relative risk than the above-mentioned E-values.

**Figure 3:**
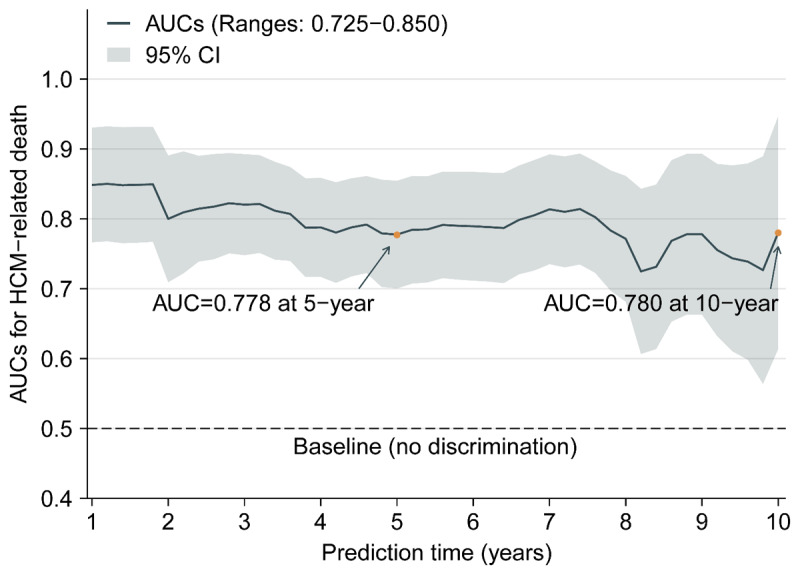
Time-dependent AUCs for ALBI score prdicting HCM-related death. *Note*: the curve was calculated every 0.2 years (from 1 to 10 years). In the figure, the solid line depicts the AUCs, and the ribbon represents 95% CI. Abbreviations as in Tables 1 and 2.

Furthermore, we assessed the discriminative power of ALBI score for HCM-related death at different time points. During the follow-up, time-dependent AUCs ranged from 0.725 to 0.850, indicating a possibly helpful, even clearly useful, discrimination of ALBI score for HCM-related death, and the predictive power was relatively stable at different time points (Figure 3). At 5-year, time-dependent AUC, best threshold of ALBI score, sensitivity and specificity were 0.778, –2.67, 70.8% and 77.5%, respectively; at 10-year, these parameters were 0.780, –2.86, 84.8% and 69.2%, respectively.

### 3.3. Comparison with other biomarkers of liver function and HCM risk-SCD score

Moreover, we performed the comparisons of ALBI score versus other parameters of liver function, including alanine aminotransferase, aspartate aminotransferase (AST), model for end-stage liver disease-XI and the components of ALBI, in predicting HCM-related death. The results displayed that only albumin and AST were associated with HCM-related death, but weaker than ALBI score (Figure 1A, Figure 3 and Table S2).

HCM risk-SCD is originally used to assess the patients with an increased risk of SCD, and the score provides individualized 5-year risk estimates [23]. There was a complete subgroup (n = 126) with the components of HCM-risk SCD score in our study; therefore, we compared ALBI score with HCM-risk SCD score in predicting HCM-related death and SCD within five years. During the follow-up, 16 patients (12.7%) reached the outcome of HCM-related death, including seven HF-related deaths, one stroke-related deaths and eight SCDs. The results showed ALBI score had a better predictive and discriminative power than HCM risk-SCD score for HCM-related death and SCD (Table S3 and Figure S3).

### 3.4. Exploratory analysis: prognostic values of the changes of ALBI score for predicting HCM-related death

**Figure 4:**
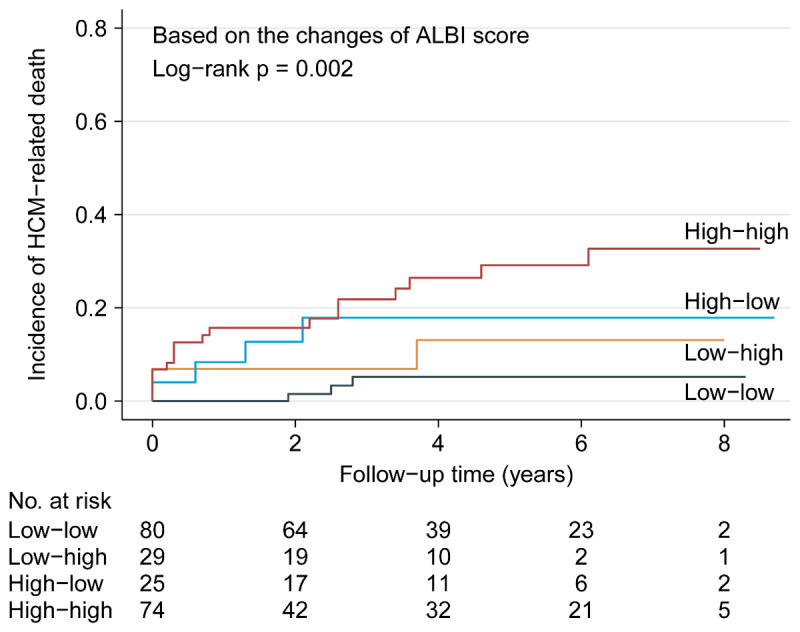
Kaplan-Meier analysis showing cumulative HCM-related death by categorical changes of ALBI score.

We also explored whether serial determinations of ALBI score could also provide useful information in predicting HCM-related death. Patients were followed for a median period of 3.6 years (IQR: 1.3–5.9 years), and 28 patients (13.5%) reached the outcome of HCM-related death after the second measurement. Based on the categorical changes, there were 80 patients in the low-low group (3 deaths, mortality rate: 3.8%), 29 in the low-high group (3 deaths, mortality rate: 10.3%), 25 in the high-low group (4 deaths, mortality rate: 16.0%) and 74 in the high-high group (18 deaths, mortality rate: 24.3%), respectively. Kaplan-Meier curve showed the clinical course was significantly worse in the high-high group, and the low-low group had the lowest mortality (log-rank p = 0.002, Figure 4). With the low-low group as reference, adjusted HRs were 2.96 for the low-high group (95% CI: 0.59–14.77, p = 0.186), 4.98 for the high-low group (95% CI: 1.11–22.37, p = 0.036) and 7.68 for the high-high group (95% CI: 2.21–26.69, p = 0.001). Due to the small number of deaths, the above analyses were only adjusted for age and gender.

### 3.5. Correlates of ALBI score with other indicators

Some variables for important pathogenetic pathways, as shown in the model 5, in HCM-related adverse outcome were also measured in our cohort. Therefore, we further analyzed the relationship between ALBI score and these variables to explore the potential mechanisms why ALBI score could predict HCM-related death. To assess the complex relationship between these variables and ALBI score, multiple linear regression model was generated based on a stepwise forward selection process (Table 3), indicating that these variables explained approximately 20.3% of the variance of ALBI score, and statistically significant correlates of ALBI score in the model were: inflammation (NLR), myocardial injury (AST), nutritional status (TG) and clinical characteristics (age, NYHA III-IV).

**Table 3:** Factors associated with ALBI score in multivariable linear models.


	β	SE OF β	p VALUE

(Intercept)	–3.074	0.170	<0.001

NLR	0.015	0.003	<0.001

TG	–0.069	0.018	<0.001

Age	0.005	0.001	<0.001

AST	0.001	0.000	0.001

NYHA III-IV	0.098	0.042	0.021

Gender	0.011	0.038	0.779

Family history of SCD	–0.161	0.103	0.118

Dyspnea	0.039	0.040	0.327

Syncope/pre-syncope	0.031	0.040	0.441

Atrial fibrillation	0.078	0.051	0.129

Urea nitrogen	0.004	0.005	0.370

LA diameter	–0.002	0.003	0.568

MWT	–0.004	0.004	0.354

Resting LVOTG >= 30 mm Hg	–0.002	0.039	0.951


*Abbreviations*: SE= standard error, other abbreviations as in Tables 1 and S1.

## 4. Discussion

In the present study, we found higher ALBI score was a strong independent predictor of HCM-related death. Our results fit into the recent series of observations on the predictive role of ALBI score in other CVDs [14,15], and extend these findings in patients with HCM. The potential mechanisms of ALBI score predicting HCM-related death may include multiple pathogenetic processes associated with HCM-related adverse outcome, including inflammation, myocardial injury, nutritional status and some clinical characteristics, but not abnormal cardiac structure and function itself. To our knowledge, this represents the first report of higher ALBI score as a potential prognostic marker for HCM-related death.

In our study, the results showed higher ALBI score could predict HCM-related death. Given the inherent biases of the retrospective study, occasionality cannot be ruled out completely. However, the high level of statistical significance was observed for HCM-related death and the relatively consistent findings across various subgroups minimize this possibility. Meanwhile, we used E-value analysis to quantify the potential implications of unmeasured confounders and found that an unmeasured confounder was unlikely to negate the risk of ALBI score for HCM-related death. Furthermore, exploratory analysis also indicated similar results. Therefore, these features support the validity of our findings.

The mechanisms underlying the association between ALBI score and HCM-related death are not clearly determined. Whereas, the present findings seemingly suggested the potential mechanisms might be out of abnormal cardiac structure and function itself, and the mechanisms may include multiple pathogenetic processes associated with HCM-related adverse outcome, including inflammation, myocardial injury, nutritional status and some clinical characteristics. Firstly, accumulating evidence suggests the existence of low-grade systemic [36,37,38] and local inflammation [18,21,22] in HCM, further resulting in adverse ventricular remodeling [22], and elevated high-sensitivity C-reactive protein has been significantly associated with increased risk of adverse outcomes in HCM [39]. Some other studies have also shown oxidative stress may be involved in the pathogenesis in HCM [19,20,40]. As a component of ALBI score, bilirubin has anti-inflammatory and antioxidant effects [12,41]; therefore, high level of bilirubin might be a reflection of increased chronic inflammation and oxidative stress. In addition, serum albumin is also a marker of inflammation and oxidative stress [42,43]. Therefore, the combination of the two indicators should be associated with inflammation and oxidative stress. In the present study, ALBI score was associated with NLR, which has been accepted as a novel marker indicating inflammation and oxidative stress [44,45], further supporting ALBI score should be a marker of inflammation and oxidative stress. Thus, based on these literature data and the novel observations of this study, inflammation and oxidative stress may be one of the potential mechanisms that ALBI score could predict HCM-related death. Secondly, recent studies have shown that cardiac troponin, an important marker of myocardial injury, is commonly elevated in HCM, and could predict the prognosis in HCM [46,47]. Myocardial injury may be caused by inappropriate cardiac hypertrophy [46] and microvascular dysfunction [48] in HCM. Currently, serum albumin and bilirubin have exerted anti-thrombotic activity [17,49] and protection of vascular endothelial cells [12,50]; therefore, abnormal serum albumin and bilirubin can deteriorate vascular function in HCM, further causing subsequent adverse outcomes. In our study, AST was designated as a marker of myocardial injury for lacking of cardiac troponin, and some studies have shown AST is associated with poor outcomes in HCM [51,52]. Our findings suggested ALBI score was associated with AST, indicating that the score may be related to myocardial injury. Therefore, myocardial injury could be another potential mechanism. Thirdly, TG is an indicator of nutritional status, and was in significant relationship with ALBI score in our study; meanwhile, albumin itself is also a marker of nutritional status. Undernutrition has been an independent prognostic factor for mortality in HCM [24]. Furthermore, ALBI score was also associated with some clinical characteristics, including age and NYHA III-IV, which can predict poor outcomes in HCM [23,30]. Taken together, it is plausible to hypothesize that ALBI score is a good integrative marker of complex clinical settings in HCM, including inflammation, myocardial injury, nutritional status and some clinical characteristics. These literature data and the novel observations of this study may help to understand why higher ALBI score is a powerful predictor for HCM-related death. Finally, the echocardiographic data was not associated with ALBI score in our study; even so, the relationship between the score and cardiac structure in HCM is still not ruled out completely. Echocardiography has inherent limitations than other imageological examinations, such as cardiac magnetic resonance (CMR), and Kuusisto J et al. [21] have described that circulatory inflammatory markers were associated with late gadolinium enhancement by CMR. Therefore, more researches are needed.

The study has several limitations. Firstly, this was a single center, retrospective study, and those patients were from China, lack of region diversification and race comparison. Secondly, since the study was performed at a tertiary referral hospital, which might have resulted in certain inherent selection biases. Thirdly, data about the drugs associated with albumin and TBil was missed. Fourthly, the study failed to perform CMR, and the measure may further help to explain the relation between ALBI score and adverse outcomes in HCM. Fifthly, the study failed to exclude patients with coronary heart disease and measure cardiac troponin, which may partly affect the exploratory analysis of potential mechanisms. Finally, we could not collect some known markers for HCM-related death, which might partly result in the biases of the results. While, E-value analysis could suggest the robustness of the findings in some degree. In spite of the above, our results are still able to clearly indicate that higher ALBI score is a risk factor of HCM-related death.

## 5. Conclusion

In summary, higher ALBI score is a strong independent predictor of HCM-related death, and the patients with higher ALBI score may warrant closer follow-up and more aggressive therapy with hopefully prevention of events in the future. Meanwhile, the present findings seemingly suggest the potential mechanisms of ALBI score predicting HCM-related death are out of abnormal cardiac structure and function itself, and the mechanisms may include multiple pathogenetic processes associated with HCM-related adverse outcome, including inflammation, myocardial injury, nutritional status and some clinical characteristics. In the future, more studies are needed to assess the association between ALBI score and outcome to better risk-stratify patients with HCM. Furthermore, it is unclear whether intervention of ALBI score, namely managing low serum albumin and high TBil, can improve the outcomes of HCM, but the exploratory analysis gives us some hints. The clinical significance of ALBI score deserves further investigations in patients with HCM.

## Data Accessibility Statement

The datasets generated during and/or analyzed during the current study are available from the corresponding author on reasonable request.

## Additional Files

The additional files for this article can be found as follows:

10.5334/gh.1163.s1 Supplementary File 1. Figures S1 to S3.

10.5334/gh.1163.s2 Supplementary File 2. Tables S1 to S3.
