# Supplementary material for: Application of the ALBI Scoring System for Mortality Outcome Prediction in Patients with Hypertrophic Cardiomyopathy
Source: Glob Heart. 2022 Oct 11;17(1):73. doi: 10.5334/gh.1163 (PMC9562974; doi:10.5334/gh.1163)

Figure S1. Relationship between ALBI score and specific modes of HCM-related death by smooth curve fitting.

Note: (A) HF-related death, (B) stroke-related death, (C) SCD. Due to the small number of specific deaths, these curves were only adjusted for age and gender. In this figure, the solid lines and ribbons depict the HRs and 95% CI, respectively.

Abbreviations as in tables 1 and 2.

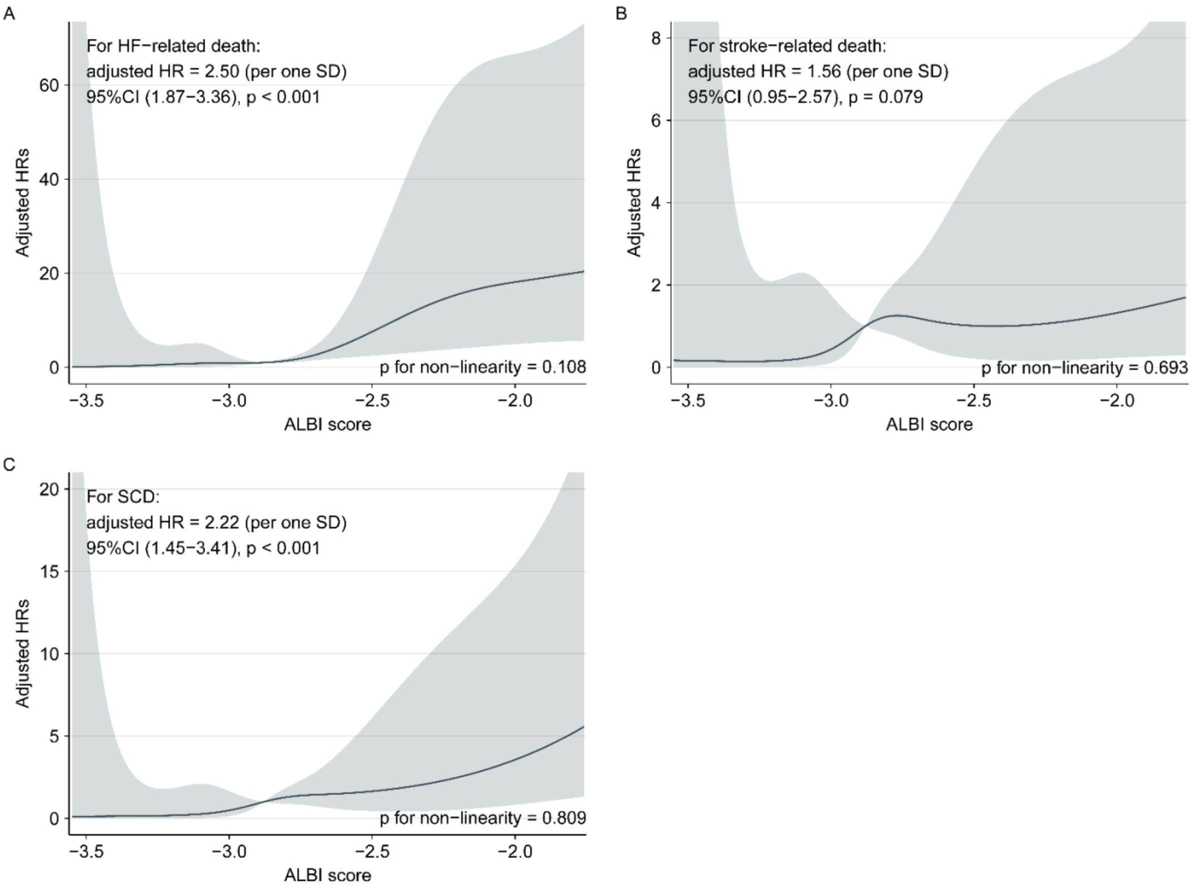

Figure S2. Kaplan-Meier curves for specific modes of HCM-related death.

Note: (A) HF-related death, (B) stroke-related death, (C) SCD.

Abbreviations as in tables 1 and 2.

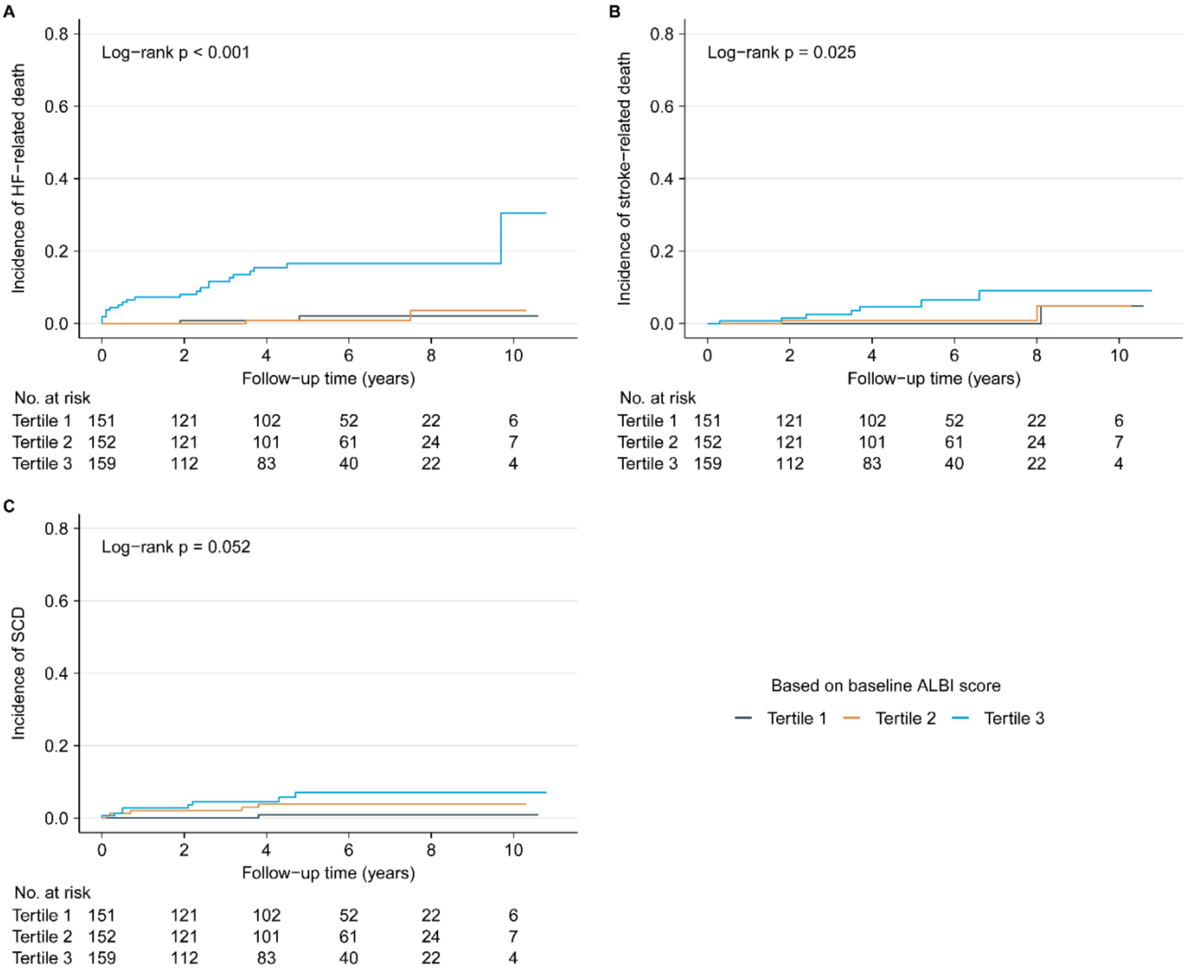

Figure S3. Time-dependent AUCs for ALBI score and HCM risk-SCD score predicting study outcome within five years.

Note: (A, B) for ALBI score, (C, D) for HCM risk-SCD score. The curves were calculated every 0.2 years (from 1 to 5 years). In the figure, the solid lines depict the AUCs, and the ribbons represent 95% CI.

Abbreviations as in tables 1 and 2

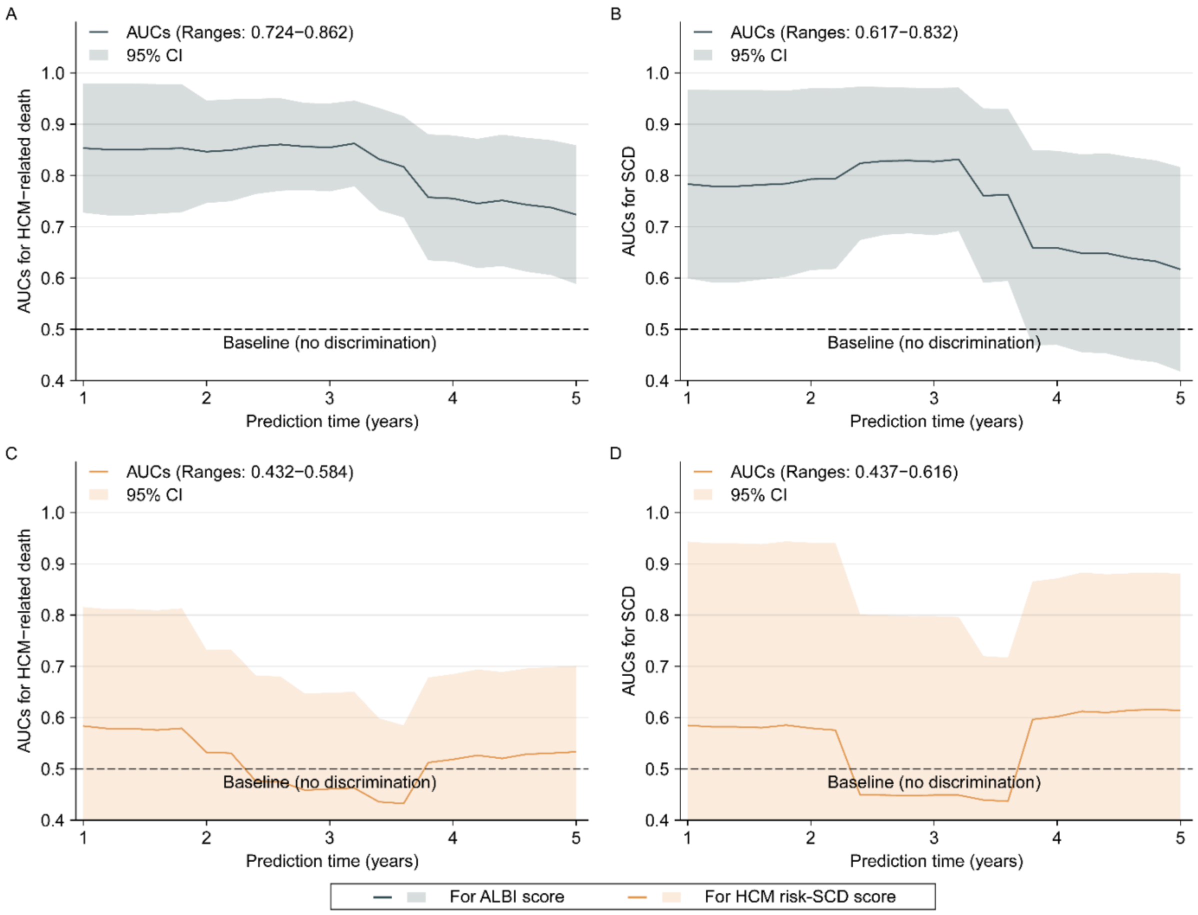

Supplement: Supplementary File 1. — Figures S1 to S3. [file gh-17-1-1163-s1.pdf]
