# Supplementary material for: Application of the ALBI Scoring System for Mortality Outcome Prediction in Patients with Hypertrophic Cardiomyopathy
Source: Glob Heart. 2022 Oct 11;17(1):73. doi: 10.5334/gh.1163 (PMC9562974; doi:10.5334/gh.1163)
Supplement: Supplementary File 2. — Tables S1 to S3. [file gh-17-1-1163-s2.pdf]

**Table S1.** Univariate Cox regression analysis for HCM-related death

| Variable | HR (95% CI) | p value |
|---|---|---|
| Gender, male | 1.35 (0.78-2.34) | 0.278 |
| Age (years) | 1.25 (0.94-1.67) | 0.128 |
| Family history of HCM | 1.15 (0.49-2.70) | 0.741 |
| Family history of SCD | 1.65 (0.51-5.31) | 0.398 |
| NYHA III-IV | 2.86 (1.65-4.96) | <0.001 |
| Dyspnea | 3.54 (1.77-7.06) | <0.001 |
| Chest pain | 0.83 (0.48-1.42) | 0.492 |
| Syncope/pre-syncope | 1.07 (0.60-1.89) | 0.826 |
| Palpitation | 0.99 (0.57-1.73) | 0.986 |
| Prior TE | 2.81 (1.20-6.58) | 0.018 |
| Vascular diseases | 1.09 (0.39-3.04) | 0.866 |
| Hypertension | 0.73 (0.39-1.37) | 0.331 |
| diabetes | 0.90 (0.32-2.49) | 0.835 |
| Atrial fibrillation | 4.10 (2.38-7.08) | <0.001 |
| Hepatic disease | 0.98 (0.35-2.72) | 0.970 |
| Aspirin | 1.05 (0.53-2.09) | 0.896 |
| Clopidogrel | 0.00 (0.00-.)† | 0.995 |
| Beta blockers | 0.98 (0.54-1.78) | 0.937 |
| ACE inhibitor or ARB | 0.73 (0.34-1.54) | 0.407 |
| Intervention of obstruction | | |
| none | 1 | |
| alcohol septal ablation | 0.21 (0.03-1.55) | 0.127 |
| septal myectomy | 1.38 (0.19-10.0) | 0.748 |
| Devices | | |
| none | 1 | |
| pacemaker | 2.41 (0.95-6.11) | 0.063 |
| ICD | 1.24 (0.49-3.13) | 0.655 |

| | | |
|---|---|---|
| ALT (IU/L) | 1.05 (0.87-1.27) | 0.632 |
| AST (IU/L) | 1.18 (1.02-1.36) | 0.026 |
| TBil (μmol/L) | 1.52 (1.26-1.84) | <0.001 |
| Albumin (g/L) | 0.50 (0.41-0.61) | <0.001 |
| Creatinine (μmol/L) | 1.05 (0.84-1.31) | 0.675 |
| Urea nitrogen (mmol/L) | 1.21 (1.06-1.38) | 0.004 |
| TG (mmol/L) | 0.54 (0.34-0.87) | 0.011 |
| HDL-C (mmol/L) | 0.90 (0.67-1.20) | 0.465 |
| LDL-C (mmol/L) | 0.76 (0.57-1.00) | 0.053 |
| Hgb (g/L) | 0.78 (0.61-1.01) | 0.059 |
| WBCC ($10^9$/L) | 1.17 (0.92-1.48) | 0.208 |
| Neutrophils count ($10^9$/L) | 1.27 (1.03-1.57) | 0.026 |
| Lymphocyte count ($10^9$/L) | 0.64 (0.46-0.88) | 0.006 |
| NLR | 1.38 (1.24-1.54) | <0.001 |
| LVEDD (mm) | 0.87 (0.64-1.19) | 0.392 |
| LA diameter (mm) | 1.53 (1.19-1.96) | 0.001 |
| MWT (mm) | 0.89 (0.68-1.18) | 0.434 |
| LVEF (%) | 0.67 (0.55-0.83) | <0.001 |
| Resting LVOTG >= 30 mm Hg | 1.14 (0.66-1.97) | 0.640 |

Note: HRs for continuous variables shown as the standardized HR (per one SD), and for categorical variables shown as the specific HRs (yes vs no), except gender (female vs male), intervention of obstruction (none as reference) and devices (none as reference).

† There was no death in patients taking clopidogrel.

Abbreviations as in tables 1 and 2.

**Table S2.** Associations of other liver parameters with HCM-related death

| Variable | Adjusted HR (95% CI), p | Ranges of time-dependent AUCs |
|---|---|---|
| ALT (IU/L) | 0.56 (0.16-1.89), 0.348 | 0.472-0.662 |
| AST (IU/L) | 1.24 (1.05-1.45), 0.010 | 0.588-0.757 |
| TBil (μmol/L) | 1.17 (0.93-1.48), 0.181 | 0.479-0.680 |
| Albumin (g/L) | 0.59 (0.45-0.77), <0.001 | 0.684-0.849 |
| MELD-XI score | 1.23 (0.81-1.86), 0.325 | 0.555-0.680 |

Note: HRs for these variables shown as the standardized HR (per one SD), and each variable was adjusted for age, gender, family history of SCD, NYHA III-IV, dyspnea, syncope/pre-syncope, atrial fibrillation, AST, urea nitrogen, TG, NLR, LA diameter, MWT and Resting LVOTG >= 30 mm Hg, except the variable itself.

MELD-XI score was calculated by: 5.11*(ln TBil [mg/dl]) + 11.76* (ln creatinine [mg/dl]) + 9.44. In addition, AUCs were calculated every 0.2 years (from 1 to 10 years).

Abbreviations: MELD-XI = model for end-stage liver disease-XI, other abbreviations as in tables 1 and S1.

**Table S3.** Associations of ALBI score and HCM Risk-SCD score with study outcome within 5 years

| Variable | Adjusted HR (95% CI), p | Ranges of time-dependent AUCs |
|---|---|---|
| For HCM-related death | | |
| ALBI score | 2.01 (1.38-2.92), p<0.001 | 0.724-0.862 |
| HCM risk-SCD score | 1.39 (0.84-2.32), 0.203 | 0.432-0.584 |
| For SCD | | |
| ALBI score | 2.12 (1.18-3.79), 0.012 | 0.617-0.832 |
| HCM risk-SCD score | 1.85 (0.95-3.57), 0.070 | 0.437-0.616 |

Note: HRs for these variables shown as the standardized HR (per one SD). Due to the small number of deaths, the analysis was only adjusted for age and gender.

HCM risk-SCD score was calculated by: $P_{SCD}$ at 5 years = $1 - 0.998^{\exp(\text{Prognostic Index})}$, where Prognostic Index = 0.15939858*MWT (mm) - 0.00294271* $MWT^2$ ($mm^2$) + 0.0259082*LA diameter (mm) + 0.00446131*Maximal LVOGT (mmHg) + 0.4583082*Family history of SCD + 0.82639195*NSVT + 0.71650361*Unexplained syncope/pre-syncope - 0.01799934*Age at clinical evaluation (years).

Abbreviations as in tables 1 and S1.
